# Calibration and validation of the Pneumonia Shock Score in critically ill patients with SARS-CoV-2 infection, a multicenter prospective cohort study

**DOI:** 10.3389/fmed.2022.958291

**Published:** 2022-08-15

**Authors:** Thomas A. Carmo, Isabella B. B. Ferreira, Rodrigo C. Menezes, Márcio L. T. Pina, Roberto S. Oliveira, Gabriel P. Telles, Antônio F. A. Machado, Tércio C. Aguiar, Juliana R. Caldas, María B. Arriaga, Kevan M. Akrami, Nivaldo M. Filgueiras Filho, Bruno B. Andrade

**Affiliations:** ^1^Universidade Salvador (UNIFACS), Salvador, Bahia, Brazil; ^2^Multinational Organization Network Sponsoring Translational and Epidemiological Research Initiative, Salvador, Brazil; ^3^Escola Bahiana de Medicina e Saúde Pública (EBMSP), Salvador, Bahia, Brazil; ^4^Faculdade de Medicina da Bahia, Universidade Federal da Bahia, Salvador, Brazil; ^5^Laboratório de Inflamação e Biomarcadores, Instituto Gonçalo Moniz, Fundação Oswaldo Cruz, Salvador, Bahia, Brazil; ^6^Hospital São Rafael, Rede Dor, Salvador, Bahia, Brazil; ^7^Divisions of Infectious Diseases and Pulmonary, Critical Care and Sleep Medicine, Department of Medicine, University of California, San Diego, La Jolla, CA, United States; ^8^Núcleo de Pesquisa, Ensino e Comunicação, Hospital de Cidade, Salvador, Bahia, Brazil

**Keywords:** critical care, prognosis, COVID-19, mortality, risk factors

## Abstract

**Background:**

Prognostic tools developed to stratify critically ill patients in Intensive Care Units (ICUs), are critical to predict those with higher risk of mortality in the first hours of admission. This study aims to evaluate the performance of the pShock score in critically ill patients admitted to the ICU with SARS-CoV-2 infection.

**Methods:**

Prospective observational analytical cohort study conducted between January 2020 and March 2021 in four general ICUs in Salvador, Brazil. Descriptive statistics were used to characterize the cohort and a logistic regression, followed by cross-validation, were performed to calibrate the score. A ROC curve analysis was used to assess accuracy of the models analyzed.

**Results:**

Six hundred five adult ICU patients were included in the study. The median age was 63 (IQR: 49–74) years with a mortality rate of 33.2% (201 patients). The calibrated pShock-CoV score performed well in prediction of ICU mortality (AUC of 0.80 [95% Confidence Interval (CI): 0.77–0.83; *p*-value < 0.0001]).

**Conclusions:**

The pShock-CoV score demonstrated robust discriminatory capacity and may assist in targeting scarce ICU resources during the COVID-19 pandemic to those critically ill patients most likely to benefit.

## Introduction

The COVID-19 outbreak created a worldwide emergency in the face of rapid dissemination throughout the world (1). To date, the pandemic has more than 240 million cases worldwide and over 4.9 million deaths spread over 220 countries (2). While most infected individuals develop mild forms of the disease, those who develop life threatening infections requiring intensive care units (ICU) care may succumb to their infection with mortality rates up to 49% (3, 4). Scarcity of healthcare resources has profoundly impacted low-middle-income countries, with significant strain on pre-existing limited ICU capacity (2, 5). In Brazil, significant viral transmissibility, associated with excess mortality rates in the elderly and those with a high burden of disease, rapidly overwhelmed health services in the country (3, 6). Existing prognostic tools to triage resources to those most likely to benefit from critical care, such as SAPS3, SOFA and APACHE IV, lack sufficient accuracy in those hospitalized with COVID-19 (7–9). Despite several novel prognostic models emerged during the pandemic, many have been found to have a high risk of bias, and not sufficient attempt has been made to develop a simple routinely applicable scoring system to early predict higher risk of mortality for patients admitted in ICUs (7). Recently, our group developed and externally validated a prognostic score for mortality risk stratification of patients admitted to the ICU with pneumonia, the Pneumonia Shock Score (pShock) (10). This tool demonstrated excellent discriminate function, outperforming other prognostic scores evaluated in our derivation and external validation cohorts. Given the severity of pneumonia in those with COVID-19, this study seeks to calibrate and evaluate the performance of the pShock score in critically ill patients admitted to the ICU with SARS-CoV-2 infection.

## Materials and methods

### Study design

This was a prospective observational analytical cohort study conducted between January 2020 and March 2021 in four general ICUs in Salvador, Bahia, Brazil. All patients older than 18 years of age with confirmed SARS-CoV-2 infection by reverse transcriptase polymerase chain reaction analysis were included. The primary outcome assessed was ICU mortality. During the study period the assistance provided at all centers was in accordance with the guidelines and protocols for COVID-19 management. Clinical and laboratory data were prospectively collected in the medical records and registered in an encrypted database stored on the RedCap system (11). Study variables included age, weight, height, sex, length of ICU and hospital stay, and physiological and laboratory data within the first 6 h of admission. Complications including need for mechanical ventilation, vasopressors, and other supportive therapy in the ICU were noted. In addition, the score derivation dataset was used to compare the performance of the original score against a calibration of the pShock score (pShock-CoV).

### Statistical analysis

Categorical variables were expressed as frequency and percentages, and continuous variables were expressed as medians with inter-quartile ranges (IQR). The proportion of categorical variables between groups were compared using Fisher's exact test. The median of continuous variables was compared using Mann-Whitney *U* test when analyzing the outcome groups. All tests were two-tailed and considered statistically significant for *p* ≤ 0.05. Variables that demonstrated possible statistical associations in univariate analysis (*p* ≤ 0.05) were transformed from continuous variables into categorical variables whose cutoff values were based on the Youden Index J on AUROC analysis. Additionally, a stepwise multivariate logistic regression was used to identify characteristics independently associated with ICU mortality. Data were categorized, then a ROC curve analysis was performed to assess accuracy and discrimination of the scores. Hosmer-Lemeshow tests for goodness of fit was used to assess the calibration of the model by comparing both the observed and expected mortality. The study was conducted accordingly with the Transparent Reporting of a multivariable prediction model for Individual Prognosis or Diagnosis (TRIPOD) guidelines (12). Extending our analyses, an internal validation using the K (10) Fold Cross Validation was performed (13). Resampling was used to evaluate the models on the data sample, using a parameter called “k” that refers to the number of groups the data sample was split into. One proportion of the data was used to discover the classification and the rest to validate and measure the prediction power of a limited data set. Probability of ICU survival during distinct timepoints since admission, was calculated by Kaplan-Meier analysis. Data analysis was carried out using GraphPad Prism version 6.01, SPSS, version 25.0 software and R statistical software.

### Ethics approval and consent to participate

The study was approved directly by the National Committee of Ethics in Research (CONEP) from Brazil in accordance with local guidelines during COVID-19 pandemic (14), Certificate of Presentation of Ethical Appreciation (CAAE) Number: 30660720.0.0000.0008, and by the Ethical Committee of the Centro de Pesquisas Gonçalo Moniz, Fundação Oswaldo Cruz (FIOCRUZ) under CAAE number 39059320.8.1001.0040. The need for informed consent was waived in both committees and the anonymity of the study subjects was preserved.

## Results

**Figure 1:**
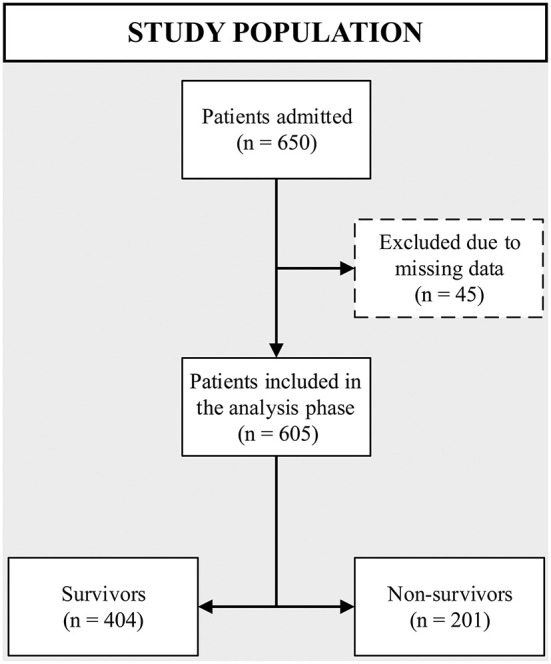
Flow chart of study enrollment and analyzed population.

**Figure 2:**
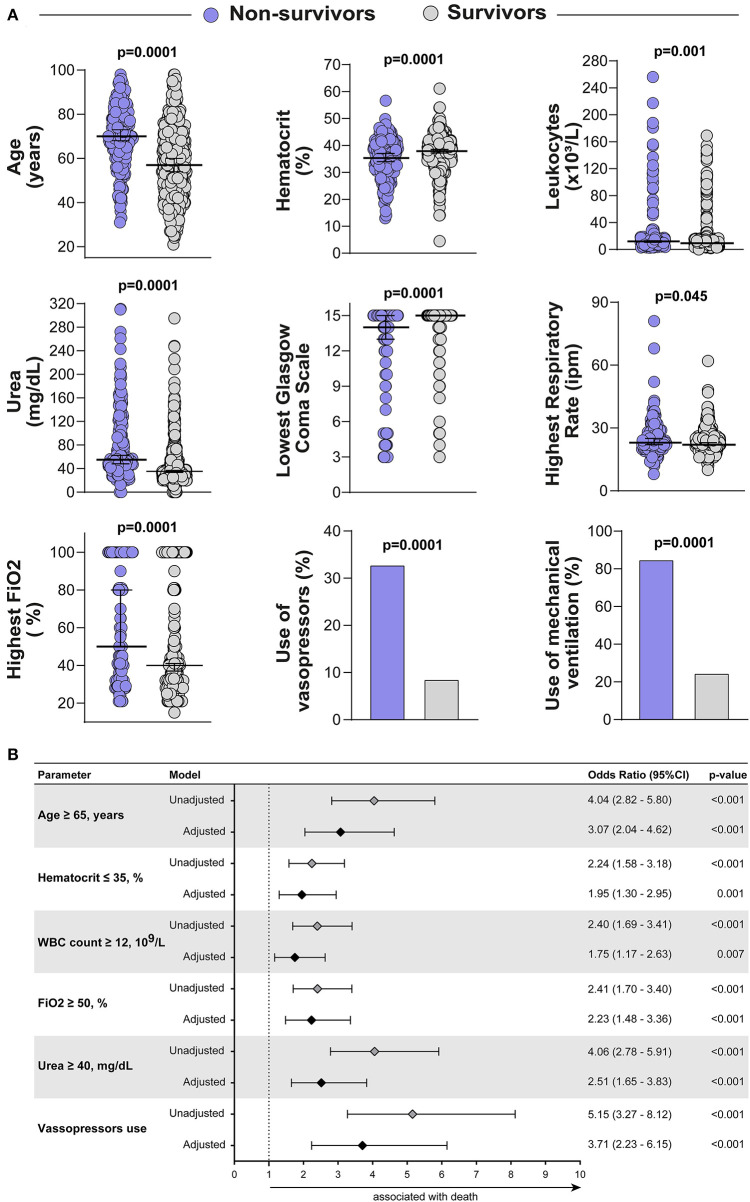
General study population description and Calibration of pShock-CoV score. **(A)** Scatter plots depicting the distribution of age, hematocrit, leukocytes, urea, lowest Glasgow coma score, highest respiratory rate and highest FiO2 in non-survivors and survivors. Lines represent median and interquartile range values. The Mann-Whitney *U* test was employed to compare the values detected between the study groups. Use of vasopressors and use of mechanical ventilation variables are shown as frequency (%) and compared using the Fisher's exact test. **(B)** Adjusted and unadjusted binary regression model for ICU mortality. Multivariable regression adjusted for differences in baseline characteristics (variables of p ≤ 0.05 identified in univariable analysis).

During the study period, 650 patients were admitted to the four study ICUs, of whom 605 met inclusion criteria (Figure 1). The median age was 63 (IQR: 49–74) years with a mortality rate of 33.2% (201 patients). Overall, non-survivors were significantly older when compared with survivors [70 (IQR: 62–80) years vs. 57 (IQR: 44–70) years; *p* ≤ 0.001]. No mortality differences were observed according to gender, nor objective clinical parameters such as heart rate, lowest systolic blood pressure, and sodium levels (Table 1). Importantly, the following factors were distinct in non-survivors compared to survivors: increased respiratory rate, elevated leukocyte count and urea, increased FiO2 within the first 6 h of admission, need for mechanical ventilation and vasopressors, and a lower Glasgow Coma Scale score and hematocrit in non survivors (Figure 2A). Description of prognostic scores analyzed are detailed in Table 2. Regarding each center characteristics, no significant discrepancies were observed concerning age distribution and vasopressors use meanwhile gender and vital signs exhibited some differences between cohorts.

**Table 1:** General population description and comparison between survivors and non survivors.

**Characteristics**	**General**	**Survivors**	**Non-survivors**	***p*-value**
	**(*n =* 605)**	**(*n =* 404)**	**(*n =* 201)**	
Age, years	63 [49–74]	57 [44–70]	70 [62–80]	**<0.001**
Male sex	366 (60)	247 (61)	119 (59)	0.647
Heart rate, beats/min	90 [79–101]	90 [79,5–100]	89 [77–103]	0.883
Respiratory rate, breaths/min	22 (20-27)	22 (20-26)	23 (20-28)	**0.045**
Systolic blood pressure, mmHg	127 [110–148]	128 [110–146]	124 [108–152]	0.601
Hematocrit, %	37,4 [32,9–40,9]	37,9 [34,3–41,3]	35,3 [30,8–40,4]	**<0.001**
Leukocytes, × 10^9^/L	9,89 [6,93–14,59]	[9,22 6,8,9,10,11,12,13,3]	11,9 [7,24–16,2]	**0.001**
Urea, mg/dL	42,6 [29–71]	35,8 [27–56,1]	55 [39–101]	**<0.001**
Sodium, mmol/L	138 [135–141]	138 [135–141]	138 [134–142]	0.661
FiO_2_, %	44 [32–100]	40 [28–100]	80 [33–100]	**<0.001**
Glasgow coma scale	15 (13-15)	15 (14,15)	14 (9-15)	**<0.001**
Use of vasopressors	101 (16.7)	35 (8.7)	66 (32.8)	**<0.001**
Mechanical ventilation	271 (45)	100 (25)	171 (85)	**<0.001**

Data are represented as median with interquartile range [25**–**75th percentile] or frequency (percentage). Clinical groups were compared using the Mann-Whitney U-test for quantitative variables and the Pearson's qui-square test or Fisher exact test for categorical variables.

FiO2, fraction of inspired oxygen.

Bold values which were statistically significant (P-value < 0.05).

**Table 2:** Prognostic scores in cohort stratified by mortality.

**Characteristics**	**General**	**Survivors**	**Non-survivors**	***p*-value**
	**(*n =* 605)**	**(*n =* 404)**	**(*n =* 201)**	
**CURB-65**				**<0.001**
0	86 (14.2)	81 (20)	5 (2.5)	
1	161 (26.6)	138 (34.2)	23 (11.4)	
2	196 (32.4)	112 (27.7)	84 (41.8)	
3	131 (21.7)	61 (15.1)	70 (34.8)	
4	30 (5)	12 (3)	18 (9)	
5	1 (0.2)	0 (0)	1 (0.5)	
**qSOFA**				**<0.001**
0	129 (21.3)	113 (28)	16 (8)	
1	305 (50.4)	199 (49.3)	106 (52.7)	
2	147 (24.3)	81 (20)	66 (32.8)	
3	24 (4)	11 (2.7)	13 (6.5)	
**pShock-CoV score**				**<0.001**
0	78 (12.9)	77 (19.1)	1 (0.5)	
1	87 (14.4)	79 (19.6)	8 (4)	
2	93 (15.4)	77 (19.1)	16 (8)	
3	109 (18)	71 (17.6)	38 (18.9)	
4	104 (17.2)	55 (13.6)	49 (24.4)	
5	70 (11.6)	27 (6.7)	43 (21.4)	
6	37 (6.1)	15 (3.7)	22 (10.9)	
7	19 (3.1)	3 (0.7)	16 (8)	
8	8 (1.3)	0 (0)	8 (4)	

Data are represented as frequency (percentage). Clinical groups were compared using the Pearson's qui-square test or Fisher exact test.

CURB-65, confusion, urea, respiratory rate, blood pressure, age; qSOFA, quick Sequential Organ Failure Assessment.

Bold values which were statistically significant (P-value < 0.05).

### pShock score development and calibration of the pShock-CoV

The original pShock score was developed in a derivation cohort of critically ill patients admitted with pneumonia in the ICU, with an external validation cohort derived from the Community-Acquired Pneumonia Organization (CAPO). The primary outcome evaluated was ICU mortality, and independent risk factors identified by a binary logistic regression were included in the composite score. Of note results were remarkable by a good prediction performance of the pShock score, with an AUC of 0.80 [95% Confidence Interval (CI): 0.73–0.86; p-value <0.0001] and better discriminate function than other models analyzed (SAPS 3, qSOFA, CURB-65, and CRB-65) (10). Further, in this study, pShock variables and clinically important parameters routinely available in the first hours of admission were assessed over multiple analyses and a stepwise multivariate logistic regression model yielded 6 variables associated with ICU mortality: age ≥ 65 years, hematocrit ≤ 35%, white blood cell count ≥ 12 × 10^9^/L, FiO2 ≥ 50%, urea ≥ 40 mg/dL and use of vasopressors (Figure 2B). The calibrated pShock-CoV score system was determined based on variability in the odds ratio for a confidence interval (CI) of 95%. Similar to the original derivation cohort for pShock, age and vasopressor use were weighted 2 points while other variables were given 1 point in the score calculation, with total score values ranging from a minimum of 0 to a maximum of 8. Notably, goodness of fit test exhibited good calibration of the model (Hosmer-Lemeshow statistics, p = 0.65).

### pShock-CoV score discrimination and validation performance

**Figure 3:**
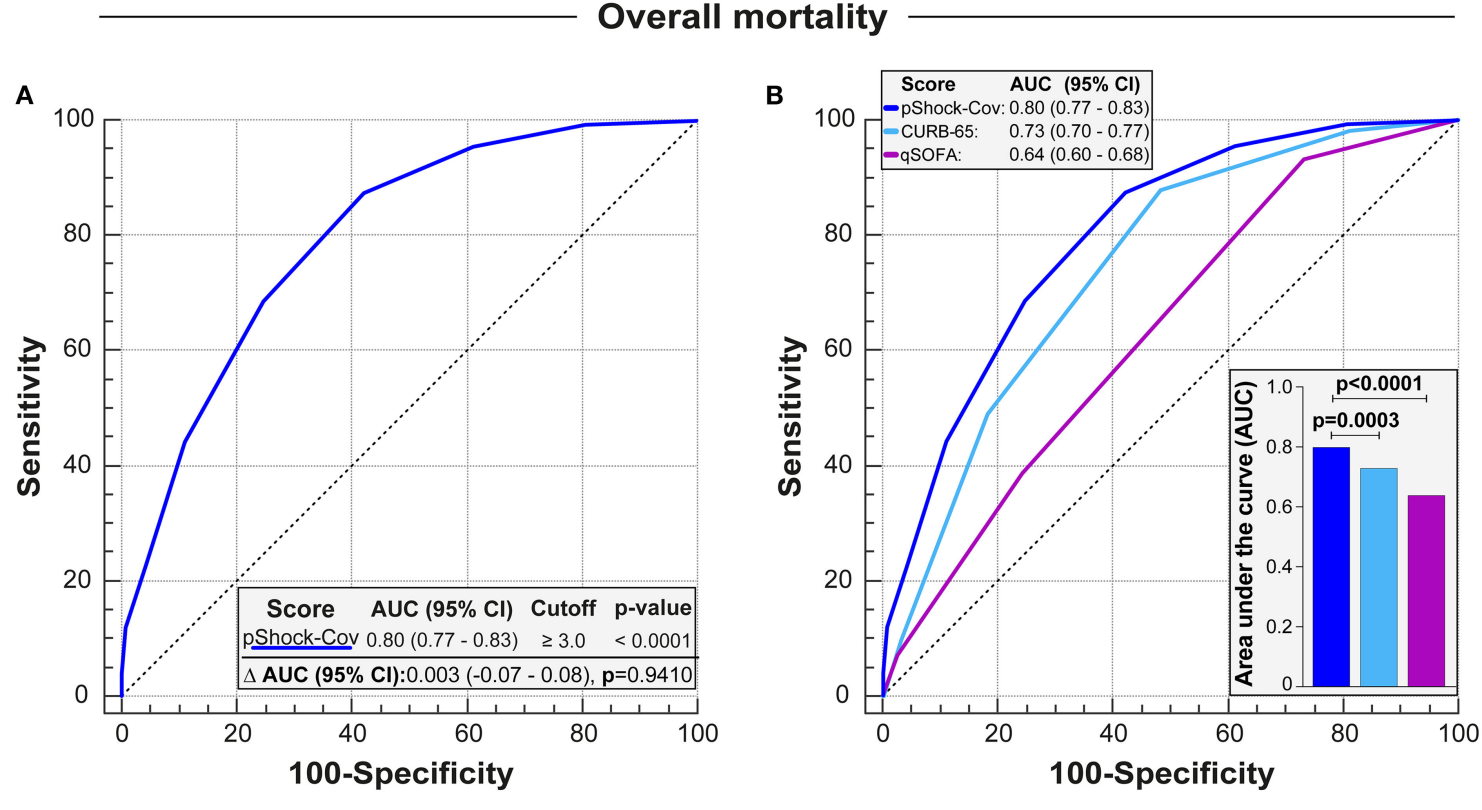
Discrimation of pShock-CoV in critically ill patients with SARS-CoV-2 infection and comparison with other severity models. **(A)** Receiver operating characteristic (ROC) curve analysis of pShock-CoV for prediction of ICU mortality in the ICU original sample and comparison of area under the ROC curve (Δ AUC) with pShock in the derivation cohort. **(B)** Overlap between ROC curves showing pShock-CoV performance and comparing with CURB-65 and qSOFA in COVID patients. Differences between AUC-ROCS were accessed by the DeLong test.

**Figure 4:**
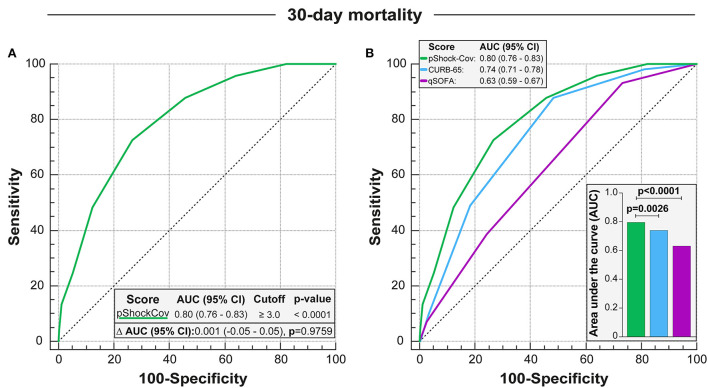
Discrimation of pShock-CoV over prediction of 30-day mortality for patients with COVID in ICU and comparison with other severity models. **(A)** Perfomance of the pShock-CoV score in predicting 30-day mortality in the intensive care unit, and comparison with discrimination capacity for overall mortality. **(B)** Comparison pShock-CoV with CURB-65 and qSOFA for prediction of ICU 30-day mortality in COVID patients. Differences between AUC-ROCS were accessed by the DeLong test.

In COVID-19 infected patients admitted to the ICU, the pShock-CoV score demonstrated robust performance accuracy with an AUC of 0.80 [95% Confidence Interval (CI): 0.77–0.83; *p*-value < 0.0001] for mortality prediction without a notable loss in discriminative capacity compared with the derivation cohort for the original pShock score (*p*-value = 0.9410, Figure 3A). The pShock-CoV score demonstrated superior discriminate function compared with CURB-65 (*p* = 0.0003) and qSOFA (*p* < 0.0001) (Figure 3B). Internal validation conducted by K (10) Fold Cross Validation analysis confirmed consistent discriminative capacity of the score compared with the original sample, with an AUC of 0.78 [95% Confidence Interval (CI): 0.71–0.83; *p*-value < 0.0001] (Supplementary Figure 1). Score performance was consistent in 30-day mortality similar to overall ICU mortality (*p*-value = 0.9759, Figure 4A), and with the others scores analyzed (Figure 4B). Temporal analysis from admission demonstrated decreased survival probability in those with higher scores of pShock-CoV over time (Supplementary Figure 2).

## Discussion

The persistence of high ICU mortality rates associated with COVID-19 infection may reflect delayed early recognition of those at highest risk of death resulting in missed opportunities to targeted interventions over the first hours of ICU admission. While ICU specific severity scores have been refined and new scores designed, a robust systematic model to predict mortality risk in a complex and diverse ICU population is lacking. Though vaccines and improved support measures have led to decreased morbidity and mortality, uncertainties remain in how best to stratify who is most likely to survive and target limited ICU resources to these patients (15). While recent studies have sought to develop prognostic tools to predict in-hospital COVID-19 mortality, these tools were not designed to evaluate risk for ICU mortality (16, 17). Other COVID-specific scores focused on triage evaluation to predict ICU admissions, which may inaccurately determine risk of deterioration and mortality in patients already admitted in these units (7, 8, 18, 19). Existing disease severity models including SOFA and SAPS3 lack adequate discriminant function, hindering accurate screening of critically ill patients in areas with supply shortages (20, 21). Furthermore, conclusions from clinical trial of novel therapeutics may be confounded as these severity scores are inaccurate in identification of the most critically ill subset of hospitalized patients with COVID-19 infection. Alternatively, to other models, pShock-CoV is a simple straightforward tool that doesn't uses radiographic images or complex variables to be obtained in the first hours of admission. In addition, some of the selected parameters are compatible with earlier described prognostic factors for COVID-19 patients, aiding the applicability of the model in routine clinical practice. While the pShock-CoV Score demonstrated significant discriminatory capacity and sustained performance in ICU and 30-day mortality including cross validation, certain study limitations must be acknowledged. First, the modest number of individuals included in the analysis may have underestimated the performance of the score in a larger ICU cohort of individuals with COVID-19 infection. Secondly, interventions including steroids, remdesivir and possibly a more experienced COVID treatment team could have impacted the performance of the score. Analysis over various time points through the pandemic demonstrated stable score performance reflecting ongoing excess mortality in those admitted to the ICU with COVID-19 independent of new treatment approaches (Supplementary Figure 3).

## Conclusions

Our calibrated pShock-CoV score is a robust bedside tool that may better define severity of disease at time of trial enrollment and ensure that results reflect the studied interventions rather than unbalanced study groups.

## Data availability statement

The raw data supporting the conclusions of this article will be made available by the authors, without undue reservation.

## Ethics statement

The studies involving human participants were reviewed and approved by National Committee of Ethics in Research (CONEP) (CAAE Number: 30660720.0.0000.0008), and by the Ethical Committee of the Centro de Pesquisas Gonçalo Moniz, Fundação Oswaldo Cruz (FIOCRUZ) (CAAE Number: 39059320.8.1001.0040). Written informed consent for participation was not required for this study in accordance with the national legislation and the institutional requirements.

## Author contributions

Conceptualization, design of study, and manuscript draft: TC, IF, RM, KA, and BA. Investigation and visualization: TC, MP, RO, GT, AM, TA, JC, and NF. Data acquisition: MP, RO, GT, AM, TA, JC, and NF. Data analysis and interpretation: TC, IF, RM, MA, KA, and BA. Supervision and critical revision: TC, IF, RM, JC, MA, KA, and BA. Editing and final approval of the manuscript: TC, RM, MA, KA, and BA. All authors read and approved the final manuscript.

## Funding

The work of BA was supported by the Intramural Research Program of the Oswaldo Cruz Foundation, Brazil. RM received a fellowship from the *Programa Nacional de Pós-Doutorado/Coordenação de Aperfeiçoamento de Pessoal de N*í*vel Superio*r (PNPD/CAPES). Fogarty International Center and National Institute of Child Health and Human Development of the National Institutes of Health under (Award Number D43 TW009763 through a research scholarship awarded to MA), MA and TC (scientific initiation with Award Number: PPSUS/BA-FAPESB003/2017/SESAB/CNPq/MS 5125/2017) received a fellowship from the *Fundação de Amparo à Pesquisa do Estado da Bahia* (FAPESB*)*.

## Conflict of interest

Authors TC, IF, RM, MA, KA, and BA were employed by fellows from Multinational Organization Network Sponsoring Translational and Epidemiological Research Initiative. The remaining authors declare that the research was conducted in the absence of any commercial or financial relationships that could be construed as a potential conflict of interest.

## Publisher's note

All claims expressed in this article are solely those of the authors and do not necessarily represent those of their affiliated organizations, or those of the publisher, the editors and the reviewers. Any product that may be evaluated in this article, or claim that may be made by its manufacturer, is not guaranteed or endorsed by the publisher.
