# Supplementary material for: Calibration and validation of the Pneumonia Shock Score in critically ill patients with SARS-CoV-2 infection, a multicenter prospective cohort study
Source: Front Med (Lausanne). 2022 Aug 15;9:958291. doi: 10.3389/fmed.2022.958291 (PMC9420902; doi:10.3389/fmed.2022.958291)
Supplement: Supplementary file 1 [file Data_Sheet_1.PDF]

Supplementary Material

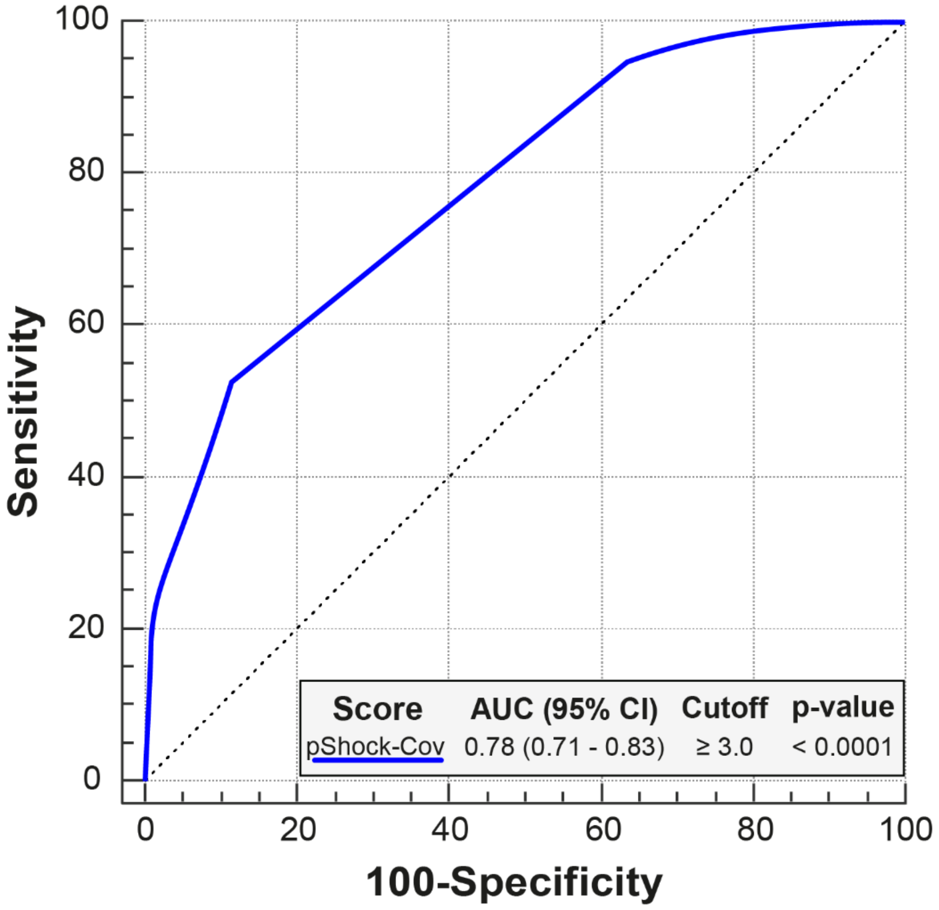

Supplementary Fig. 1. Internal validation of pShock-Cov using the K (10) resampling analysis. ROC curve after K-10 fold-validation, showing model accuracy.

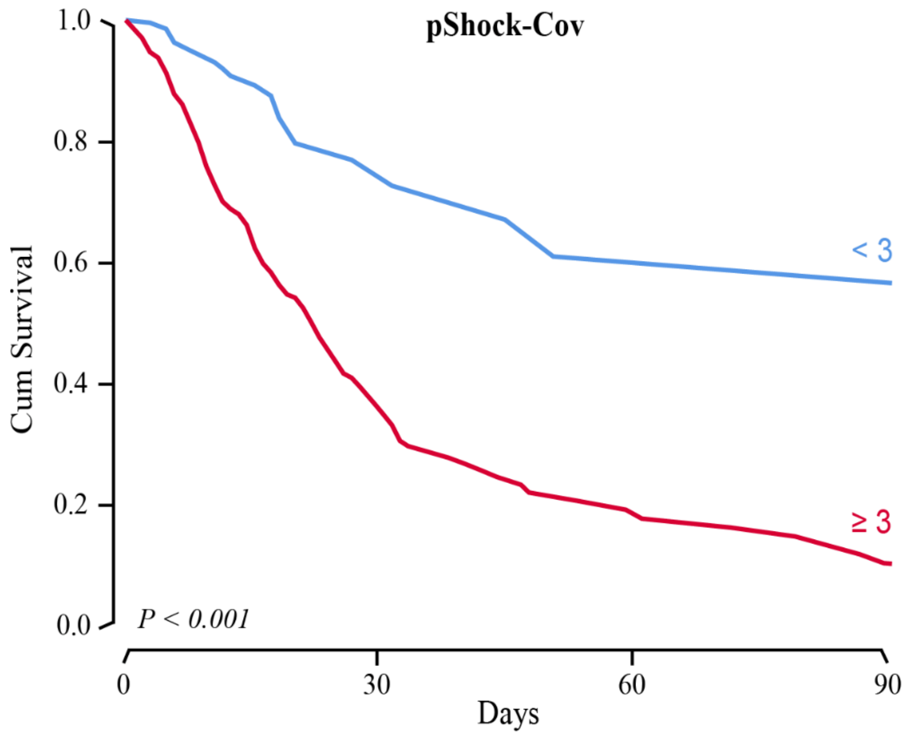

Number at Risk

pShock-Cov < 3	252	22	8	7
pShock-Cov ≥ 3	344	51	14	10

Supplementary Fig. 2. Probability of ICU survival over time stratified by pShock-Cov cutoffs points. Modified Kaplan-Meier analysis for an pShock-Cov cutoff of 3 points.

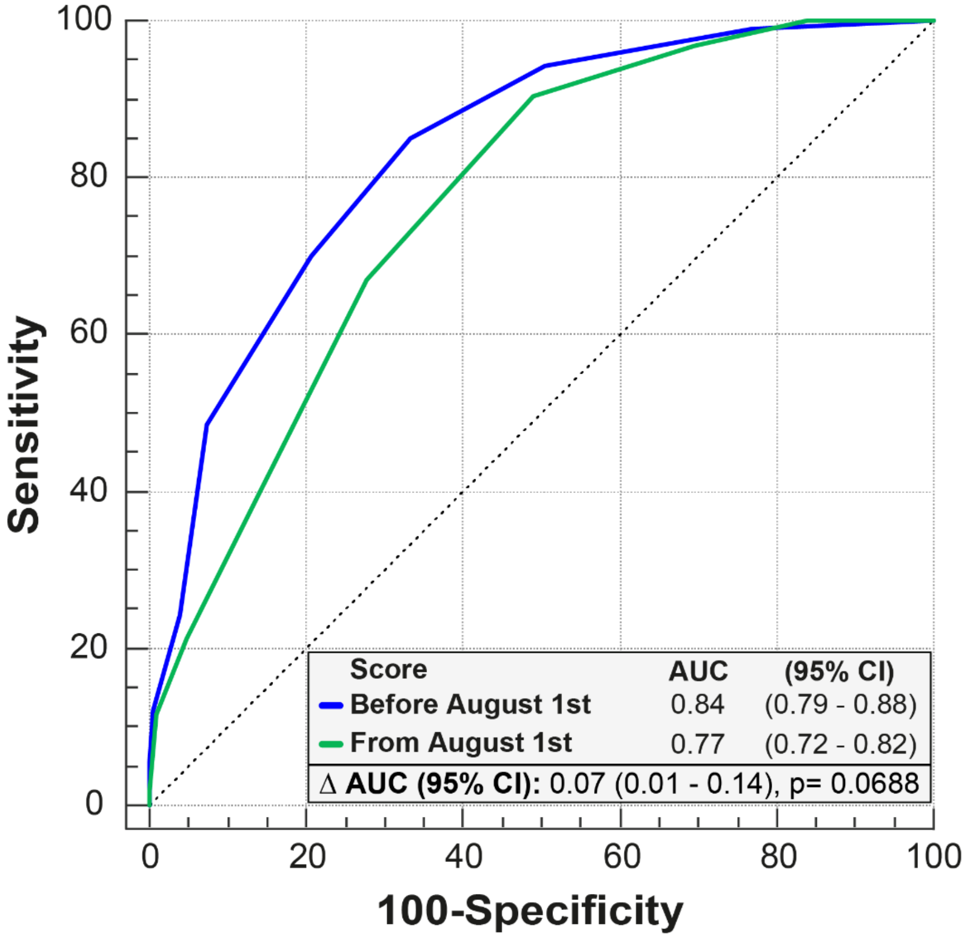

Supplementary Fig. 3. Performance of pShock-Cov score over distinct timepotins. ROC curve analysis of pShock-Cov for ICU mortality prediction before and from August 1st of 2021.
